# The medium latency muscle response to a vestibular perturbation is increased after depression of the cerebellar vermis

**DOI:** 10.1002/brb3.782

**Published:** 2017-08-22

**Authors:** Chris K. Lam, William R. Staines, Craig D. Tokuno, Leah R. Bent

**Affiliations:** ^1^ University of Guelph Guelph ON Canada; ^2^ University of Waterloo Waterloo ON Canada; ^3^ Brock University St. Catharines ON Canada

**Keywords:** cerebellum, continuous theta burst stimulation, galvanic vestibular stimulation, reflexes, vestibular

## Abstract

**Introduction:**

Galvanic vestibular stimulation (GVS) is able to evoke distinct responses in the muscles used for balance. These reflexes, termed the short (SL) and medium latency (ML) responses, can be altered by sensory input; decreasing in size when additional sensory cues are available. Although much is known about these responses, the origin and role of the responses are still not fully understood. It has been suggested that the cerebellum, a structure that is involved in postural control and sensory integration, may play a role in the modulation of these reflexes.

**Methods:**

The cerebellar vermis was temporarily depressed using continuous theta burst stimulation and SL, ML and overall vestibular electromyographic and force plate shear response amplitudes were compared before and after cerebellar depression.

**Results:**

There were no changes in force plate shear amplitude and a non‐significant increase for the SL muscle response (*p* = .071), however, we did find significant increases in the ML and overall vestibular muscle response amplitudes after cerebellar depression (*p* = .026 and *p* = .016, respectively). No changes were evoked when a SHAM stimulus was used.

**Discussion:**

These results suggest that the cerebellar vermis plays a role in the modulation of vestibular muscle reflex responses to GVS.

## 1 INTRODUCTION

Sensory input from the vestibular apparatus is essential for maintaining postural stability and can be manipulated by passing an electrical current through the mastoid processes (galvanic vestibular stimulation; GVS). GVS alters the firing of the peripheral vestibular afferents and elicits the sensation of imbalance, which consequently evokes a postural sway response (Britton et al., 1993; Coats & Stoltz, 1969; Fitzpatrick, Burke, & Gandevia, 1994; Lund & Broberg, 1983). GVS also produces a well‐documented reflex response in the muscles that are used to maintain balance. The typical response is observed in the soleus muscle as a biphasic modulation comprised of two distinct parts, a short latency (SL; 40–70 ms after stimulus onset) response and a medium latency (ML; 70–140 ms) response (Britton et al., 1993).

The ML response has been shown to be related to the postural sway (Britton et al., 1993; Fitzpatrick et al., 1994) and can be modulated by sensory input such as vision and somatosensory feedback (Britton et al., 1993; Lund & Broberg, 1983; Muise, Lam, & Bent, 2012; Welgampola & Colebatch, 2001). Welgampola and Colebatch (2001) found a decrease in the magnitude of the ML response when they provided vision or enabled the use of additional somatosensory feedback (touch). Similarly, Muise et al. (2012) found the ML response was modulated with altered sensory input. These authors elicited a reduction in cutaneous feedback from the plantar foot sole and observed an increase in the ML response. Together this work demonstrates the strong influence of sensory input on the modulation of the ML response. In contrast, the function of the SL is not as fully understood. One group of researchers has shown the response magnitude could be decreased by additional sensory input (Welgampola & Colebatch, 2001), however, the majority agree that the SL response remains unchanged across conditions of increased or decreased sensory contribution (Britton et al., 1993; Fitzpatrick et al., 1994; Muise et al., 2012).

There has been much discussion around the nature of the SL and ML responses, including the specific origin of the responses (e.g., semicircular canals or otolith organs); whether they have a common descending pathway (e.g., vestibulospinal or reticulospinal tract) and if the responses are modulated by other cortical structures (Britton et al., 1993; Cathers, Day, & Fitzpatrick, 2005; Fitzpatrick et al., 1994; Mian, Dakin, Blouin, Fitzpatrick, & Day, 2010). Based on the latency of the ML response, and its ability to be modified by sensory input, it has been hypothesized that this vestibular reflex response could be modulated by the cerebellum (Cathers et al., 2005), a structure where sensory input converges for comparison and integration (Apps & Garwicz, 2005; Chadderton, Schaefer, Williams, & Margrie, 2014; Wiestler, McGonigle, & Diedrichsen, 2011).

In contrast, it is less likely that the SL response is modulated by the cerebellum, first based on its latency (~40–70 ms), which does not provide time to travel through cerebellar pathways before descending to the muscle and second, there is a lack of evidence showing the SL is actually modulated by sensory input. As a result, it is postulated that the SL response is not processed by the cerebellum but is an unaltered response transmitted directly to the muscle via vestibulospinal pathways.

The main objective of this study was to examine if the cerebellum, and in particular the vermis (center of sensory integration and postural control), modulates the reflexive responses to a vestibular perturbation. Using transcranial magnetic stimulation (TMS), we temporarily depressed the function of the vermis and examined changes to the reflexive muscle activity of the soleus and the shear forces under the feet. It was hypothesized that the SL response would remain unchanged, but the posturally relevant ML response would be modulated after depression of the cerebellum. In particular we expected the ML response amplitude to increase, as output from the cerebellum is inhibitory.

## 2 METHODS

### 2.1 Subjects

Twelve adults (six males and six females) between the ages of 20–28 years (mean ± 1 standard deviation of 23 ± 2.3 years) with no history of neurological or musculoskeletal disorders participated in the study. Written informed consent was obtained from all subjects prior to data collection. The experimental procedures were approved by the Research Ethics Board at the University of Guelph and the Office of Research Ethics at the University of Waterloo and conformed to the standards set by the Declaration of Helsinki.

### 2.2 Experimental overview

The study consisted of two separate sessions, a test session which involved the use of continuous theta burst stimulation TMS to induce cerebellar depression and a SHAM session, which utilized paired pulse TMS. Each subject took part in both sessions, randomized and separated by at least a week. For all trials, participants stood barefoot with their feet together (<1 cm intermalleolar distance), head facing forward and eyes closed. During each trial subjects were perturbed using GVS, which was delivered during six 8 min blocks of 100 stimulations. Three blocks were administered before and three more after the application of TMS. Reflexive EMG responses and force plate shear forces were measured during GVS trials. For the application of TMS, subjects were seated and TMS was administered to the back of the head over the cerebellar vermis. TMS consisted of either continuous theta burst stimulation (TEST; to induce cerebellar depression: see below) or as paired pulse stimulation (SHAM; to not induce depression).

### 2.3 Experimental setup

EMG data were collected from the soleus muscles bilaterally (gain 1000, band pass filtered between 10 and 1000 Hz; Bortec AMT‐8 system, Bortec Biomedical Ltd, Alberta, Canada). Skin overlaying each muscle was cleaned with alcohol wipes and two silver, silver‐chloride electrodes (Ag/AgCl; Meditrace Mini, King Medical, King City, Canada) were placed lengthwise, 3 cm apart on the skin over the muscle belly.

Force plate shear data were collected on a multi‐axis force plate (Advanced Mechanical Technology Inc., MA, USA). Both EMG and shear data were collected using a custom designed Labview program (version 2012 SP1; RRID: SCR_014325; National Instruments, Austin TX, USA) and were digitized at 2000 Hz.

### 2.4 Galvanic vestibular stimulation

The skin over the mastoids was first cleaned with alcohol wipes then electrically conductive gel was added between the electrodes and skin to lower the impedance. Two Ag/AgCl electrodes (Meditrace Mini, King Medical, King City, Canada) were adhered to the mastoid processes to deliver bipolar, binaural vestibular stimulation. The current was delivered via a constant current isolated stimulator (model A395, World Precision Instruments, Florida, USA). Prior to testing, each subject was assessed for individual GVS threshold. GVS threshold was determined by administering a low level current to standing subjects with their eyes closed. Stimulus intensity began at approximately 0.1 mA and was gradually increased in increments of 0.05 mA until a slight head tilt was observed accompanied by a slight sway of the body toward the anode electrode (Bent, McFadyen, French Merkley, Kennedy, & Inglis, 2000). During testing, GVS (500 ms square wave pulse) was delivered to subjects at three times threshold with a minimum current of 1.5 mA (range: 1.5–1.8 mA). GVS was applied in a random polarity [anode right cathode left (AR), cathode right anode left (CR)] once every 4–6 s in sets of 100 stimuli. Six sets (3 pre (approximately 30 min) and 3 post TMS intervention) of 100 stimuli were collected for a total of 600 GVS pulses. The head orientation was controlled by having the participants focus on a target at eye level on the wall in front of them prior to the onset of each set. Experimenters monitored the head throughout the set and informed the participants to reorient their head if it moved. Breaks were provided between each set where the subject was allowed to open their eyes and sit down.

### 2.5 Transcranial magnetic stimulation

#### 2.5.1 Threshold

Subjects underwent TMS threshold testing on the same day of testing for each protocol. For the test protocol (cTBS), a MagPro X100 magnetic stimulator (MCF‐B65 Butterfly Coil, MagVenture Inc. GA, USA) was used for both threshold and cTBS application, and for the SHAM protocol, a figure‐of‐8 coil (Double 70 mm Remote Control Coil, Magstim BiStim2 stimulator, Magstim, UK) was used for threshold and paired pulse stimulation. Participants always wore earplugs (ULINE Bullet Earplugs, Uline Canada, ON, Canada) when receiving TMS to protect their ears from the loud click sound as the stimulator discharged. Subjects were seated with their right arm resting comfortably on a table in front of them. Two Ag/AgCl electrodes were placed on the skin over their right first dorsal interosseous muscle (FDI); one over the muscle belly and the other at the metacarpal‐phalangeal joint. In order to determine active motor threshold (AMT), single pulse magnetic stimuli were delivered over the FDI representative area of the left motor cortex (approximately 6 cm lateral and 3 cm anterior to the vertex). AMT was defined as the stimulus intensity when only five out of 10 motor evoked potentials reached an amplitude of 200 μV during a 10% maximal contraction. Brainsight2 technology (Rogue Research Inc, Montreal, Canada) was utilized to ensure the location and orientation of the TMS coil remained consistent relative to the subject's head.

#### 2.5.2 Test protocol

Once threshold was determined, the subject rested their forehead on a pillow on the table, which exposed the back of their head to allow for stimulation of the cerebellum. TMS was applied to the cerebellar vermis, located directly beneath the inion [located by palpating the base of the skull, (Hashimoto & Ohtsuka, 1995)] with the coil held tangentially to the scalp with the handle pointing upwards. Subjects received continuous theta burst stimulation (cTBS), a pattern of repetitive TMS (bursts of 3 pulses of 50 Hz stimulation, repeated at 5 Hz (every 200 ms), for a total of 600 stimuli over 40 seconds (Huang, Edwards, Rounis, Bhatia, & Rothwell, 2005). cTBS was administered at each subject's 100% AMT, as described above (Demirtas‐Tatlidede, Freitas, Pascual‐Leone, & Schmahmann, 2011).

#### 2.5.3 Sham protocol

The SHAM protocol involved the application of paired pulse stimulation (2 pulses 10 ms apart) over the cerebellar vermis at 50% AMT. The SHAM parameters were designed to minimize the effects elicited with the TMS. This aspect of the study was included to ensure that effects elicited by cTBS were indeed due to cerebellar depression and not habituation to the vestibular stimulus or behavioral changes.

### 2.6 Data analysis

#### 2.6.1 Electromyography

**Figure 1:**
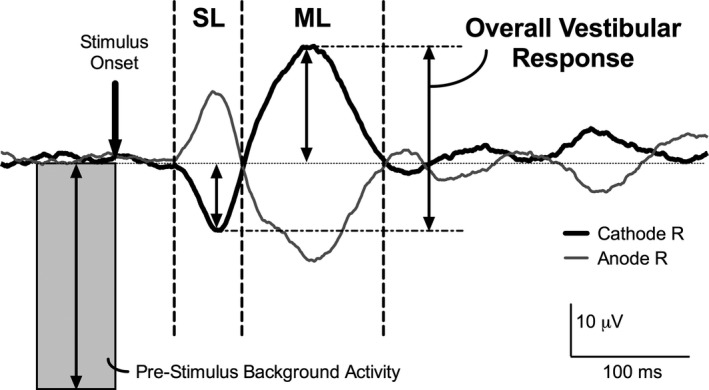
Rectified, smoothed and spike triggered averaged trace of muscle activity averaged over all subjects (*n* = 11). Cathode right and Anode right responses were overlaid to facilitate response detection. The shaded area represents the pre‐stimulus background activity used to normalize response amplitudes and the thick downward arrow represents the stimulus onset. The first response occurring at approximately 40–70 ms is the short latency response, labeled SL, the latter response, labeled ML is the medium latency response starting at the termination of the SL response (approximately 70–120 ms) and the overall vestibular response (peak of SL to peak of ML) is demonstrated to the right of the ML response. Double arrow lines represent amplitude measurement. Vertical dotted lines represent onsets and offsets of the responses, determine by visual inspection, when traces diverged from each other or the baseline

Raw EMG were rectified and filtered using a moving average with a 30 ms window. Data were then spike trigger averaged to the onset of the vestibular stimulus of the same polarity (600 ms window; 100 ms before and 500 ms after stimulus onset). Finally, data were zeroed to the mean amplitude of 50 ms preceding stimulus onset (Figure 1).

AR and CR EMG traces were overlaid for the identification of the SL and ML vestibular responses (Watson & Colebatch, 1997; Welgampola & Colebatch, 2001). The onsets of the responses were defined as the point when AR and CR traces clearly deviated from each other and deviated from the baseline between 40–70 ms (for SL) and 70–140 ms (for ML) (Figure 1). Response magnitudes were quantified as the mean absolute amplitude (averaged 10 ms around the peak) of the zeroed traces. Values were normalized to the average background EMG activity 50 ms before stimulus onset of non‐zeroed data (Muise et al., 2012; Watson & Colebatch, 1997; Welgampola & Colebatch, 2001). In addition to separating the vestibular response into distinct SL and ML responses, an overall vestibular response was measured, which was the difference between the peak of the SL response to the peak of the ML response (Figure 1) (Son, Blouin, & Inglis, 2008).

It was found that responses from the muscle contralateral to the cathodal stimulus were larger than those ipsilateral to the cathodal stimulus, similar to results observed with respect to head orientation (Britton et al., 1993; Watson & Colebatch, 1998; Welgampola & Colebatch, 2001), therefore, the response from the left soleus from CR stimuli and the response from the right soleus from AR stimuli were averaged together (total: 300 stimuli for pre and 300 stimuli for post) to assess the response to the vestibular stimulation across trials (Rosengren & Colebatch, 2002; Watson & Colebatch, 1997). The effects of TMS were assessed by comparing the normalized pre condition SL and ML amplitudes (TEST or SHAM) to their corresponding post condition amplitudes.

#### 2.6.2 Shear forces

Force plate shear represents the net response of all contributing muscles and have been correlated with the SL and ML EMG responses (Marsden, Castellote, & Day, 2002). Raw shear force data in the frontal plane were spike trigger averaged to the onset of the vestibular stimulus (1100 ms window—100 ms before and 1,000 ms after stimulus onset). Data were zeroed to the average amplitude of the background activity 50 ms before stimulus onset. AR and CR traces were overlaid and distinct shear force responses were evident beginning at approximately 100 ms and 200 ms, and are proposed to be directly related to the SL and ML responses seen in the muscles (Marsden et al., 2002). The peak amplitude of the SL and ML shear force (averaged over 10 ms), which occurred around 150 and 450 ms, respectively, were measured and averaged.

### 2.7 Statistical analysis

Background EMG amplitude (50 ms duration before stimulus onset) was assessed using a student's paired *t* test comparing soleus activity pre and post TMS. This was to ensure background EMG levels were constant across conditions, as background EMG has been shown to influence the size of the SL and ML responses (Fitzpatrick et al., 1994; Mian & Day, 2014). Effects of cerebellar cTBS were assessed using a two‐way repeated measures analysis of variance (ANOVA) to test the effects of “TMS” (TEST or SHAM) and “Time” (PRE or POST) on the dependent variables SL and ML EMG amplitude, and shear force amplitude. Normality and sphericity of response amplitudes were evaluated using Shapiro–Wilk and Mauchley's tests, respectively. If data were not normally distributed, data were log transformed and any further outliers were removed for statistical analysis. Force plate shear data for one participant was removed because after log transformation it was still greater than 3 SD above the mean. Upon removal of this outlier, the data were normally distributed. Significant effects were followed up with a priori pairwise comparisons. All statistical analyses were performed using SPSS version 22 (RRID: SCR_002865; IBM Corp. Armonk NY, USA). Significance was set to *p* < .05.

## 3 RESULTS

Eleven of twelve subjects produced quantifiable SL and ML reflex responses to GVS in the soleus muscles. As a result, data from eleven subjects were included in further analyses. The onset latencies of the SL and ML responses were within the range set by our defining criteria (SL: 41–75 ms and ML: 88–128 ms) and reflex amplitudes were comparable to other studies even with the head facing forward as opposed to over the shoulder, which is the orientation more commonly used to evoke vestibular reflexes (Britton et al., 1993; Muise et al., 2012; Nashner & Wolfson, 1974; Watson & Colebatch, 1997; Welgampola & Colebatch, 2001). SL amplitudes ranged from 1.7% to 23.2% of background EMG and ML amplitudes ranging from 3.1% to 29.1% of background (Welgampola & Colebatch, 2001; : (SL: 1%–36.1%; ML: 3.5%–25.9%), Muise et al., 2012 (SL: 3.7%–18.9%; ML: 5.8%–58.8%).

### 3.1 Background EMG

To ensure consistent levels of EMG activation, the background level of EMG activity was assessed pre and post TMS application using student's paired t‐tests. There was no statistical difference in EMG level between pre (36.4 ± 3.92 μV) and post (37.9 ± 3.96 μV) TEST condition (*p* = .135) or SHAM condition (pre: 39.3 ± 2.99 μV; post: 41.0 ± 3.34 μV, *p* = .205).

### 3.2 Short latency

**Figure 2:**
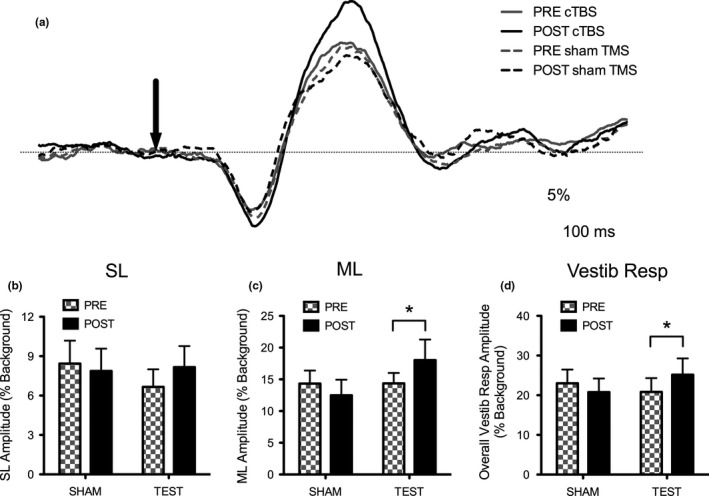
(a) Cumulative average of all contralateral EMG responses (*n* = 11) before and after TMS. The solid traces represent the test condition and the dotted traces are the SHAM condition. The gray traces represent the SL and ML responses before TMS and the black traces are the SL and ML responses after TMS. The downward arrow is the onset of the electrical stimulus. (b) Normalized short latency reflex amplitudes (% of background activity) of pre (black) and post (checkered) TMS for both TEST and SHAM conditions. (c) Normalized medium latency reflex amplitudes (% of background activity) of pre (black) and post (checkered) TMS for both TEST and SHAM conditions. (d) Normalized overall vestibular response amplitudes (peak of SL to peak of ML; % of background activity) of pre (black) and post (checkered) TMS for both TEST and SHAM conditions. Error bars represent standard error and significance is represented with a * when *p* < .05

A significant interaction existed for TMS by Time (*F*_(1,9)_ = 8.381, *p* = .016) for SL reflex response amplitude. A priori planned comparisons determined that although it was not significant, there was a trend of increased amplitude between the pre and post for the TEST condition (pre: 6.66% ± 1.34%, post: 8.17% ± 1.60%; *t*_(1,10)_ = −2.016, *p* = .071). There was no significant difference between pre and post for the SHAM condition (pre: 8.44% ± 1.78%, post: 7.87% ± 1.70%; *t*_(1,10)_ = 0.827, *p* = .427) (Figure 2a and b). Tukey post hoc analysis was performed on the remaining comparisons and found no other significant differences (*p* > .05).

### 3.3 Medium latency

A significant interaction was found for TMS by Time (*F*_(1,10)_ = 10.324, *p* = .009). A priori planned comparisons indicated that there was a significant increase in ML amplitude from 14.16% ± 2.43% to 17.21% ± 3.16% from before to after cTBS (TEST pre to TEST post) (*t*_(1,10)_ = −2.617, *p* = .026). Tukey post hoc analysis was performed on the remaining comparisons and found no other significant differences (*p* > .05). In particular, for the SHAM condition, no difference in amplitude was found from pre to post SHAM stimulation (pre: 14.53% ± 2.11%; post: 13.29% ± 2.03%; *t*_(1,10)_ = 1.176, *p* = .267) (Figure 2a and c).

### 3.4 Overall vestibular response

A significant interaction was found for TMS by Time (*F*_1,10_ = 26.768, *p* = .0004). A priori planned comparisons indicated that there was a significant increase in the overall vestibular response amplitude from 20.84% ± 3.47% to 25.13% ± 4.12% from before to after cTBS (TEST pre to TEST post) (*t*_(1,10)_ = −2.881, *p* = .016). Tukey post hoc analysis was performed on the remaining comparisons and found no other significant differences (*p* > .05). In particular, for the SHAM condition, no difference in amplitude was found from pre to post SHAM stimulation (pre: 23.02% ± 3.46%; post: 20.74% ± 3.45%; t_(1,10)_ = 1.507, *p* = .163) (Figure 2a and d).

### 3.5 Shear forces

**Figure 3:**
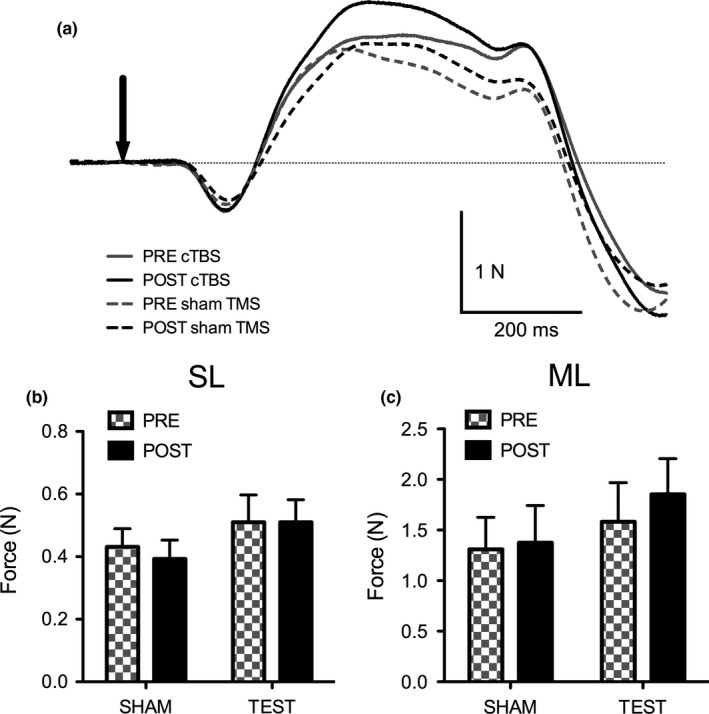
(a) Cumulative average of all shear responses (*n* = 11) before and after TMS. Responses are displayed in a single direction for clarity. The solid traces represent the test condition and the dotted traces are the SHAM condition. The gray traces represent the shear response before TMS and the black traces represent the shear after TMS. The downward arrow is the onset of the electrical stimulus. (b and c) SL and ML Fx shear force (N) of pre (black) and post (checkered) TMS for both TEST and SHAM conditions. Error bars represent standard error

There were no significant main effects for TMS (*F*_(1,10)_ = 0.748, *p* = .407) or Time (*F*_(1,10)_ = 0.500, *p* = .496) nor was there an interaction for TMS by Time (*F*_(1,10)_ = 0.581, *p* = .463) for the SL shear response. The SL amplitude for the TEST condition remained unchanged from before to after cTBS (0.51 ± 0.29 N to 0.51 ± 0.24 N) and decreased, although not significantly, from 0.43 ± 0.19 N to 0.39 ± 0.19 N during the SHAM condition (Figure 3a and b).

The ML shear response amplitudes ranged from 0.24 N to 2.303 N in the TEST condition and from 0.197 N to 2.268 N in the SHAM condition. Data from one subject was removed for statistical analysis, as it was more than 3 SD above the mean after log transformation (shear amplitude response of 9.249 N). There were no significant main effects for TMS (*F*_(1,9)_ = 1.046, *p* = .333) or Time (*F*_(1,9)_ = 2.211, *p* = .171), nor was there an interaction for TMS by Time (*F*_(1,9)_ = 3.078, *p* = .113). During the TEST conditions the shear amplitudes showed non‐significant increases from 1.58 ± 0.15 N to 1.85 ± 0.19 N after cTBS and from 1.31 ± 0.26 N to 1.37 ± 0.27 N during the SHAM condition (Figure 3a and c).

## 4 DISCUSSION

In this study, we demonstrated that the cerebellum plays a role in the modulation of the reflexive muscle responses to a vestibular perturbation. With no significant changes in background EMG activity, we saw a significant increase in the amplitude of the ML response and the overall vestibular response. Although non‐significant, there was also a trend for an increase in the SL amplitude, which could lead us to believe that the cerebellum might potentially modulate this response as well.

In this study we showed that the overall vestibular response (measured as a peak of SL response to the peak of the ML response) was modulated following a decrease in activity at the level of the cerebellum. More specifically, we showed that the ML response to a vestibular perturbation was significantly affected by depression of the cerebellar vermis. We believe this indicates that the cerebellum has a modulatory role in the development of this specific part of the postural response to a vestibular perturbation. While changes evoked from cTBS were small, with an average increase in ML amplitude of 3.16% (corresponding to a 21% increase from baseline), these effects are comparable in magnitude to sensory modulation shown in other studies: (Welgampola and Colebatch (2001) showed a 25%–60% decrease in ML amplitude with the addition of visual and somatosensory input, and Muise et al. (2012) found that after reduction in skin feedback from the plantar foot sole, the amplitude of the ML increased by 36%); and more importantly, these effects were not induced in the SHAM TMS condition.

While an increase in the vestibular response amplitude was hypothesized after cerebellar depression, only a moderate change was expected as the mechanism of cTBS is believed to be synaptic depression rather than a complete block of function.

### 4.1 The ML is significantly modulated, but the SL is not as clear

The origin and function of the EMG responses to GVS are still not fully understood. It has been proposed that the SL and ML reflexes are generated independently and are transmitted via different pathways (reticulospinal (SL) vs. vestibulospinal (ML) tracts) (Britton et al., 1993; Cathers et al., 2005; Fitzpatrick et al., 1994; Marsden et al., 2002). There has also been some speculation that the cerebellum might be involved in the development of these responses (Cathers et al., 2005; Manzoni, Pompeiano, Bruschini, & Andre, 1999) since areas of the cerebellum, such as the vermis and the flocculonodular lobe, have significant roles in processing sensory input (Morton & Bastian, 2004; Sprague & Chambers, 1953). It has been shown that the postural response to a vestibular perturbation is abnormal with cerebellar depression (Lam, Tokuno, Staines, & Bent, 2016) and in cerebellar patients compared to healthy individuals (Kammermeier, Kleine, Eggert, Krafczyk, & Büttner, 2013), which also supports a cerebellar role in the vestibular response. It was, however, unknown whether the reflexive muscle responses (SL and ML responses, and the overall vestibular response) were affected under conditions of cerebellar dysfunction/modulation and also if SL and ML responses would be affected differently.

Our data showed a significant change in the ML response but only a trend in SL amplitude after cerebellar depression from cTBS. This supports our hypothesis of a role for the cerebellum in the ML response, however, it is still remains uncertain what effect the cerebellum has on the SL response. A post hoc analysis found the effect size to be 0.309 and underpowered (power = 0.2) to detect a change in the SL response. There is the possibility that the cerebellum does not modulate the SL response, but rather, the response involves more direct pathways. However, based on experiments involving cortical stimulation, the latency of the motor evoked potentials in the soleus is approximately 30 ms (Beck et al., 2007; Ziemann, Netz, & Homberg, 1993), which is at least 10 ms sooner than the earliest onset of the SL response. Therefore, it is a strong possibility that there is sufficient time, and that the cerebellum does indeed affect the response, particularly since our data showed that there was a trend for an increase. Even if the SL response does not travel through the cerebellum, it is likely the cerebellar output is still able to modulate the excitability of the vestibular nuclei, which would result in a change in SL response amplitude (Forbes et al., 2016). We may not have been able to detect significant changes in the SL response due to certain parameters of the experimental protocol, including the intensity of the stimulus and the postural orientation. With the stimulation intensity of 1.5–1.8 mA, the SL response was quite variable, ranging from 1.7% to 23.2% of background EMG. There is literature to suggest that the SL response may not be consistent or reliable when the vestibular stimulus is below 2 mA (Ali, Rowen, & Iles, 2003; Fitzpatrick et al., 1994). Additionally, the quality of the reflexes may have been compromised with head orientation, as the reflexes are obtainable [as evidenced by the results of our study and other experiments (Dakin, Son, Inglis, & Blouin, 2007; Day et al., 1997; Forbes et al., 2016; Nashner & Wolfson, 1974)], yet not as prominent in the soleus muscle with the head facing forward (Fitzpatrick et al., 1994). Most studies examining vestibular reflexes have subjects stand with their head facing over the shoulder (Britton et al., 1993; Fitzpatrick et al., 1994; Lund & Broberg, 1983; Nashner & Wolfson, 1974; Welgampola & Colebatch, 2001), however, we opted to test the reflexes with head forward as we have shown in a previous experiment that depressing the cerebellar vermis with cTBS can result in a modulation in the postural sway direction, such that it is no longer intra‐aural with the head facing over the shoulder (Lam et al., 2016). We believe this would have led to confounding results in the current work, with alterations in the magnitude of the soleus burst. Additionally, a reduced anterior‐posterior sway effect has also been shown in cerebellar patients when their head is facing over their shoulder (Kammermeier et al., 2013). Presently, we are unable to ascertain whether the SL muscle response is definitively affected by the cerebellum due to non‐significant results as well as the limitations to our protocol.

### 4.2 Force plate shear responses

Post cTBS, there was an increase in the cumulative shear force toward the anode. However, unlike the effects observed with the ML response, changes were not significant. Shear forces reflect changes in muscular activity transmitted to the ground, and Marsden et al. (2002) related the biphasic shear response evoked by GVS to the reflexive SL and ML muscle responses. The first inflection in shear force we observed, occurring at approximately 100 ms, corresponded to the SL response, and was not modulated after cerebellar depression. The second response, with an average onset of 200 ms, trended toward an increase in amplitude after cTBS, similar to the ML EMG response, however, did not reach significance.

In this experiment, we examined the soleus muscles bilaterally, which is a plantar flexor muscle that generates movement primarily in the anteroposterior direction. Our GVS responses were evoked with a mediolateral inter‐aural axis, eliciting mediolateral sway. During an inverted pendulum full body postural response there will undoubtedly be other muscles such as the tibialis anterior, medial and lateral gastrocnemii and peronei muscles, which will actively contribute to the ankle torque and ultimately to the cumulative shear forces measured in the mediolateral direction. During postural recovery from GVS, the force responses to GVS are very sensitive to the instantaneous equilibrium state. In other words, depending on where the subjects are in their sway when perturbed by the vestibular stimulus, deviating from or returning to equilibrium, the level and involvement of different muscles will affect the size of the shear responses (Marsden et al., 2002). Alterations in the size of the soleus EMG response can be controlled through normalization of the response amplitudes to the background activity prior to stimulus onset, however, since shear force does not have offset background activity, there is no similar value to normalize to.

### 4.3 Did we depress the vermis?

The goal of the study was to examine whether partial depression of the cerebellum could modulate the response to a vestibular perturbation. Continuous theta burst stimulation (cTBS) was used to depress the function of the vermis region of the cerebellum. cTBS, when applied to the motor cortex as well as the cerebellum, has been shown to inhibit the output of neurons through what is believed to be a depletion of neurotransmitter (Huang et al., 2005; Koch et al., 2008; Popa, Russo, & Meunier, 2010). In the cerebellum, it is postulated that the depressive effects take place at the synapse between Purkinje cells and climbing fibers, parallel fibers or mossy fibers, inhibiting Purkinje output, and thus decreasing the inhibitory effects on the deep cerebellar nuclei. The effect of cTBS on cerebellar output was not directly quantified in this study, however, it has been demonstrated previously through stimulation of the lateral cerebellum (Koch et al., 2008; Popa et al., 2010). Researchers were able to activate the cerebellothalamocortical circuit and were able to show that cTBS successfully suppressed cerebellar output through a disinhibition of a motor evoked potential (Koch et al., 2008; Popa et al., 2010). Previous work has indicated that the neuronal makeup of the cerebellum is homologous between regions (Ito, 1984) and therefore we suggest that as long as the depth of the target area is controlled for (Demirtas‐Tatlidede et al., 2010), cTBS should have the effect of output depression when applied to the vermis.

The SHAM stimulus does not have the same depressive effects as cTBS based on the profile and intensity of stimulation. Paired pulse TMS is generally used to examine the effects of excitatory or inhibitory pathways in the motor cortex and the effects last less than a second (Chen, 2000; Chen et al., 2008). All participants were naive to the technique of TMS and its outcomes. After participating in both sessions, they were asked about both techniques, and although they were aware of the physical differences (duration and intensity of stimulation and tactile sensation), they were not aware that there might be a difference in the underlying effects.

## 5 CONCLUSION

We found that temporary depression of the cerebellum using cTBS modulated the vestibular muscle reflexes, significantly increasing the size of the ML response and overall vestibular response and showing a trend in the SL response. This supports the hypothesis that the cerebellum plays a role in modulation of the posturally relevant ML response, however, it remains uncertain its effects on the SL response. Previously, the origin of SL and ML responses were irresolute. Our work may shed some light on the debate, and may open some avenues for further investigations into the distinction between the two responses. Additional research is also warranted to further investigate how cerebellar depression can influence other aspects of the vestibulospinal reflex, including its specific role in sensory integration and how the response changes under various sensory conditions. Overall, we have demonstrated that the cerebellum plays a significant role in the modulation of the electromyographic responses to a vestibular stimulus.

## CONFLICT OF INTEREST

None declared.

## References

[brb3782-bib-0001] Ali, A. S. , Rowen, K. A. , & Iles, J. F. (2003). Vestibular actions on back and lower limb muscles during postural tasks in man. Journal of Physiology, 546, 615–624. https://doi.org/10.1113/jphysiol.2002.030031 12527747 10.1113/jphysiol.2002.030031 PMC2342524

[brb3782-bib-0002] Apps, R. , & Garwicz, M. (2005). Anatomical and physiological foundations of cerebellar information processing. Nature Reviews Neuroscience, 6, 297–311. https://doi.org/10.1038/nrn1646 15803161 10.1038/nrn1646

[brb3782-bib-0003] Beck, S. , Taube, W. , Gruber, M. , Amtage, F. , Gollhofer, A. , & Schubert, M. (2007). Task‐specific changes in motor evoked potentials of lower limb muscles after different training interventions. Brain Research, 79, 51–60. https://doi.org/10.1016/j.brainres.2007.08.048 10.1016/j.brainres.2007.08.048 17889840

[brb3782-bib-0004] Bent, L. R. , McFadyen, B. J. , French Merkley, V. , Kennedy, P. M. , & Inglis, J. T. (2000). Magnitude effects of galvanic vestibular stimulation on the trajectory of human gait. Neuroscience Letters, 279, 157–160. https://doi.org/10.1016/S0304-3940(99)00989-1 10688053 10.1016/s0304-3940(99)00989-1

[brb3782-bib-0005] Britton, T. C. , Day, B. L. , Brown, P. , Rothwell, J. C. , Thompson, P. D. , & Marsden, C. D. (1993). Postural electromyographic responses in the arm and leg following galvanic vestibular stimulation in man. Experimental Brain Research, 94, 143–151. https://doi.org/10.1007/BF00230477 8335069 10.1007/BF00230477

[brb3782-bib-0006] Cathers, I. , Day, B. L. , & Fitzpatrick, R. C. (2005). Otolith and canal reflexes in human standing. Journal of Physiology, 563, 229–234. https://doi.org/10.1113/jphysiol.2004.079525 15618274 10.1113/jphysiol.2004.079525 PMC1665570

[brb3782-bib-0007] Chadderton, P. , Schaefer, A. T. , Williams, S. R. , & Margrie, T. W. (2014). Sensory‐evoked synaptic integration in cerebellar and cerebral cortical neurons. Nature Reviews Neuroscience, 15, 71–83. https://doi.org/10.1038/nrn3648 24434910 10.1038/nrn3648

[brb3782-bib-0008] Chen, R. (2000). Studies of human motor physiology with transcranial magnetic stimulation. Muscle and Nerve, 23(S9), S26–S32. 10.1002/1097-4598(2000)999:9<::aid-mus6>3.0.co;2-i 11135281

[brb3782-bib-0009] Chen, R. , Cros, D. , Curra, A. , Di Lazzaro, V. , Lefaucheur, J. P. , Magistris, M. R ., … Ziemann, U . (2008). The clinical diagnostic utility of transcranial magnetic stimulation: Report of an IFCN committee. Clinical Neurophysiology, 119, 504–532. https://doi.org/10.1016/j.clinph.2007.10.014 18063409 10.1016/j.clinph.2007.10.014

[brb3782-bib-0010] Coats, A. C. , & Stoltz, M. S. (1969). The recorded body‐sway response to galvanic stimulation of the labyrinth: A preliminary study. Laryngoscope, 79, 85–103. https://doi.org/10.1288/00005537-196901000-00004 4303843 10.1288/00005537-196901000-00004

[brb3782-bib-0011] Dakin, C. J. , Son, G. M. L. , Inglis, J. T. , & Blouin, J.‐S. (2007). Frequency response of human vestibular reflexes characterized by stochastic stimuli. Journal of Physiology, 583, 1117–1127. https://doi.org/10.1113/jphysiol.2007.133264 17640935 10.1113/jphysiol.2007.133264 PMC2277188

[brb3782-bib-0012] Day, B. L. , Séverac Cauquil, A. , Bartolomei, L. , Pastor, M. A. , & Lyon, I. N. (1997). Human body‐segment tilts induced by galvanic stimulation: A vestibularly driven balance protection mechanism. Journal of Physiology, 500(Pt 3), 661–672. 9161984 10.1113/jphysiol.1997.sp022051 PMC1159417

[brb3782-bib-0013] Demirtas‐Tatlidede, A. , Freitas, C. , Cromer, J. R. , Safar, L. , Ongur, D. , Stone, W. S ., … Pascual‐Leone, A . (2010). Safety and proof of principle study of cerebellar vermal theta burst stimulation in refractory schizophrenia. Schizophrenia Research, 124, 91–100. https://doi.org/10.1016/j.schres.2010.08.015 20817483 10.1016/j.schres.2010.08.015 PMC3268232

[brb3782-bib-0014] Demirtas‐Tatlidede, A. , Freitas, C. , Pascual‐Leone, A. , & Schmahmann, J. D. (2011). Modulatory effects of theta burst stimulation on cerebellar nonsomatic functions. Cerebellum, 10, 495–503. https://doi.org/10.1007/s12311-010-0230-5 21132574 10.1007/s12311-010-0230-5 PMC3260524

[brb3782-bib-0015] Fitzpatrick, R. , Burke, D. , & Gandevia, S. C. (1994). Task‐dependent reflex responses and movement illusions evoked by galvanic vestibular stimulation in standing humans. Journal of Physiology, 478(Pt 2), 363–372. 7965852 10.1113/jphysiol.1994.sp020257 PMC1155693

[brb3782-bib-0016] Forbes, X. P. A. , Luu, B. L. , Van Der Loos, X. H. F. M. , Croft, E. A. , Inglis, J. T. , & Blouin, J. S. (2016). Transformation of vestibular signals for the control of standing in humans. Journal of Neuroscience, 36, 11510–11520. https://doi.org/10.1523/jneurosci.1902-16.2016 27911755 10.1523/JNEUROSCI.1902-16.2016 PMC6601712

[brb3782-bib-0017] Hashimoto, M. , & Ohtsuka, K. (1995). Transcranial magnetic stimulation over the posterior cerebellum during visually guided saccades in man. Brain, 118(Pt 5), 5–1193. https://doi.org/10.1093/brain/118.5.1185 10.1093/brain/118.5.1185 7496779

[brb3782-bib-0018] Huang, Y.‐Z. , Edwards, M. J. , Rounis, E. , Bhatia, K. P. , & Rothwell, J. C. (2005). Theta burst stimulation of the human motor cortex. Neuron, 45, 201–206. https://doi.org/10.1016/j.neuron.2004.12.033 15664172 10.1016/j.neuron.2004.12.033

[brb3782-bib-0501] Ito, M . (1984). The cerebellum and neural control.

[brb3782-bib-0019] Kammermeier, S. , Kleine, J. F. , Eggert, T. , Krafczyk, S. , & Büttner, U. (2013). Disturbed vestibular‐neck interaction in cerebellar disease. Journal of Neurology, 260, 794–804. https://doi.org/10.1007/s00415-012-6707-z 23081756 10.1007/s00415-012-6707-z

[brb3782-bib-0020] Koch, G. , Mori, F. , Marconi, B. , Codecà, C. , Pecchioli, C. , Salerno, S. , … Caltagirone, C . (2008). Changes in intracortical circuits of the human motor cortex following theta burst stimulation of the lateral cerebellum. Clinical Neurophysiology, 119, 2559–2569. https://doi.org/10.1016/j.clinph.2008.08.008 18824403 10.1016/j.clinph.2008.08.008

[brb3782-bib-0021] Lam, C. K. , Tokuno, C. D. , Staines, W. R. , & Bent, L. R. (2016). The direction of the postural response to a vestibular perturbation is mediated by the cerebellar vermis. Experimental Brain Research, 234, 3689–3697. https://doi.org/10.1007/s00221-016-4766-6 27601251 10.1007/s00221-016-4766-6

[brb3782-bib-0022] Lund, S. , & Broberg, C. (1983). Effects of different head positions on postural sway in man induced by a reproducible vestibular error signal. Acta Physiologica Scandinavica, 117, 307–309. https://doi.org/10.1111/j.1748-1716.1983.tb07212.x 6603098 10.1111/j.1748-1716.1983.tb07212.x

[brb3782-bib-0023] Manzoni, D. , Pompeiano, O. , Bruschini, L. , & Andre, P. (1999). Neck input modifies the reference frame for coding labyrinthine signals in the cerebellar vermis: A cellular analysis. Neuroscience, 93, 1095–1107. https://doi.org/10.1016/S0306-4522(99)00275-4 10473274 10.1016/s0306-4522(99)00275-4

[brb3782-bib-0024] Marsden, J. F. , Castellote, J. , & Day, B. L. (2002). Bipedal distribution of human vestibular‐evoked postural responses during asymmetrical standing. Journal of Physiology, 542, 323–331. https://doi.org/10.1113/jphysiol.2002.019513 12096073 10.1113/jphysiol.2002.019513 PMC2290383

[brb3782-bib-0025] Mian, O. S. , Dakin, C. J. , Blouin, J.‐S. , Fitzpatrick, R. C. , & Day, B. L. (2010). Lack of otolith involvement in balance responses evoked by mastoid electrical stimulation. Journal of Physiology, 588, 4441–4451. https://doi.org/10.1113/jphysiol.2010.195222 20855437 10.1113/jphysiol.2010.195222 PMC3008850

[brb3782-bib-0026] Mian, O. S. , & Day, B. L. (2014). Violation of the craniocentricity principle for vestibularly evoked balance responses under conditions of anisotropic stability. Journal of Neuroscience, 34, 7696–7703. https://doi.org/10.1523/jneurosci.0733-14.2014 24872573 10.1523/JNEUROSCI.0733-14.2014 PMC4035529

[brb3782-bib-0027] Morton, S. M. , & Bastian, A. J. (2004). Cerebellar control of balance and locomotion. Neuroscience, 10, 247–259. https://doi.org/10.1177/1073858404263517 10.1177/1073858404263517 15155063

[brb3782-bib-0028] Muise, S. B. , Lam, C. K. , & Bent, L. R. (2012). Reduced input from foot sole skin through cooling differentially modulates the short latency and medium latency vestibular reflex responses to galvanic vestibular stimulation. Experimental Brain Research, 218, 63–71. https://doi.org/10.1007/s00221-012-3002-2 22278107 10.1007/s00221-012-3002-2

[brb3782-bib-0029] Nashner, L. M. , & Wolfson, P. (1974). Influence of head position and proprioceptive cues on short latency postural reflexes evoked by galvanic stimulation of the human labyrinth. Brain Research, 67, 255–268. https://doi.org/10.1016/0006-8993(74)90276-5 4470421 10.1016/0006-8993(74)90276-5

[brb3782-bib-0030] Popa, T. , Russo, M. , & Meunier, S. (2010). Long‐lasting inhibition of cerebellar output. Brain Stimulation, 3, 161–169. https://doi.org/10.1016/j.brs.2009.10.001 20633445 10.1016/j.brs.2009.10.001

[brb3782-bib-0031] Rosengren, S. M. , & Colebatch, J. G. (2002). Differential effect of current rise time on short and medium latency vestibulospinal reflexes. Clinical Neurophysiology, 113, 1265–1272. https://doi.org/12140006 12140006 10.1016/s1388-2457(02)00121-9

[brb3782-bib-0032] Son, G. M. L. , Blouin, J.‐S. , & Inglis, J. T. (2008). Short‐duration galvanic vestibular stimulation evokes prolonged balance responses. Journal of Applied Physiology, 105, 1210–7. https://doi.org/10.1152/japplphysiol.01398.2006 18669937 10.1152/japplphysiol.01398.2006

[brb3782-bib-0033] Sprague, J. M. , & Chambers, W. W. (1953). Regulation of posture in intact and decerebrate cat: I. cerebellum, reticular formation vestibular nuclei. Journal of Neurophysiology, 16, 451–463. 13097194 10.1152/jn.1953.16.5.451

[brb3782-bib-0034] Watson, S. R. , & Colebatch, J. (1997). EMG responses in the soleus muscles evoked by unipolar galvanic vestibular stimulation. Electroencephalography and Clinical Neurophysiology and Motor Control, 105, 476–483. https://doi.org/10.1016/S0924-980X(97)00044-1 10.1016/s0924-980x(97)00044-1 9448650

[brb3782-bib-0035] Watson, S. R. , & Colebatch, J. G. (1998). Vestibular‐evoked electromyographic responses in soleus: A comparison between click and galvanic stimulation. Experimental Brain Research, 119, 504–10. https://doi.org/10.1007/s002210050366 9588785 10.1007/s002210050366

[brb3782-bib-0036] Welgampola, M. S. , & Colebatch, J. G. (2001). Vestibulospinal reflexes: Quantitative effects of sensory feedback and postural task. Experimental Brain Research, 139, 345–353. https://doi.org/10.1007/s002210100754 11545473 10.1007/s002210100754

[brb3782-bib-0037] Wiestler, T. , McGonigle, D. J. , & Diedrichsen, J. (2011). Integration of sensory and motor representations of single fingers in the human cerebellum. Journal of Neurophysiology, 105, 3042–3053. https://doi.org/10.1152/jn.00106.2011 21471398 10.1152/jn.00106.2011

[brb3782-bib-0038] Ziemann, U. , Netz, A. S. , & Homberg, V. (1993). Spinal and supraspinal mechanisms contribute to the silent period in the contracting soleus muscle after transcranial magnetic stimulation of human motor cortex. Neuroscience Letters, 156, 167–171. 8414181 10.1016/0304-3940(93)90464-v

